# Sitting Interruption Modalities during Prolonged Sitting Acutely Improve Postprandial Metabolome in a Crossover Pilot Trial among Postmenopausal Women

**DOI:** 10.3390/metabo14090478

**Published:** 2024-08-30

**Authors:** Jeffrey S. Patterson, Brinda K. Rana, Haiwei Gu, Dorothy D. Sears

**Affiliations:** 1 College of Health Solutions, Arizona State University, 850 N. 5th Street, Phoenix, AZ 85004, USA; jspatte5@asu.edu (J.S.P.);; 2 Department of Psychiatry, UC San Diego, La Jolla, CA 92093, USA; 3 Department of Family Medicine, UC San Diego, La Jolla, CA 92093, USA; 4 Department of Medicine, UC San Diego, La Jolla, CA 92093, USA; 5 UCSD Moores Cancer Center, 3855 Health Sciences Dr, La Jolla, CA 92093, USA

**Keywords:** postprandial metabolism, sedentary behavior, 5-hydroxynorvaline, aspartate, anserine, raffinose, aging, endothelial function, blood flow, glycemic control

## Abstract

Older adults sit during most hours of the day; more than 30% are considered physically inactive. The accumulation of prolonged sitting time is an exercise-independent risk factor for aging-related conditions such as cardiometabolic disease and cancer. Archival plasma samples from a randomized controlled, four-condition crossover study conducted in 10 postmenopausal women with overweight or obesity were analyzed. During 5-hour conditions completed on separate days, the trial tested three interruption modalities: two-minute stands each 20 min (STS), hourly ten-minute standing breaks (Stand), hourly two-minute walks (Walk), and a controlled sit. Fasting baseline and 5-hour end point (2 h postprandial) samples were used for targeted metabolomic profiling. Condition-associated metabolome changes were compared using paired *t*-tests. STS eliminated the postprandial elevation of amino acid metabolites that was observed in the control. A norvaline derivative shown to have anti-hypertensive and -hyperglycemic effects was significantly increased during Stand and STS. Post-hoc testing identified 19 significantly different metabolites across the interventions. Tight metabolite clustering by condition was driven by amino acid, vasoactive, and sugar metabolites, as demonstrated by partial least squares-discriminant analyses. This exploratory study suggests that brief, low-intensity modalities of interrupting prolonged sitting can acutely elucidate beneficial cardiometabolic changes in postmenopausal women with cardiometabolic risk.

## 1. Introduction

More than two million U.S. women reach postmenopausal status annually, and this population represents a group with significant aging-related health risk [1]. Moreover, almost one-third of this population reports no physical activity outside of the workplace, potentially due to prevalent factors related to bone health, muscle wasting, and social and environmental barriers [2]. Sedentary behavior is commonly associated with prolonged hourly sitting, which is significantly correlated with chronic cardiometabolic disease and cancer risk [3,4,5]. This risk is heightened in postmenopausal women due to age-related hormonal and metabolic alterations and increases the prevalence of having one or two chronic conditions in this population [6,7,8]. The deleterious effects of menopause place women at an increased risk for conditions like dyslipidemia, inflammation, glucose intolerance, insulin resistance, and type 2 diabetes (T2D) [9]. Hormonal changes like diminished estrogen and elevated androgen increase susceptibility to altered energy metabolism and abdominal obesity [10]. The lipolysis of visceral adipose tissue results in raised circulating free fatty acids, which have been shown to increase insulin resistance risk [11].

Sitting for extended periods during the day causes significant lower extremity vascular impairment [12,13]. Prolonged sitting bouts trigger an elevation in hydrostatic pressure and reduced hemodynamics as a product of ninety-degree arterial bending at the hip and knee joints [13]. Associated cardiovascular disease (CVD) is accompanied by macrovascular and microvascular dysfunction and circulating metabolite imbalances resulting in atherosclerosis [13]. As a result, extensive uninterrupted sitting time is commonly reported along with several chronic diseases and a risk factor for CVD [3,4,5]. In addition, it has been shown that two hours of daily MVPA is needed to counteract these negative effects of long sitting bouts [14].

Feasible and effective methods to reduce periods of prolonged sitting and associated cardiometabolic incidence are needed to improve postmenopausal health. Rising to stand immediately increases lower extremity circulation and shear stress on vascular endothelial cells that improves intercellular and intracellular signaling and lowers oxidative stress [15,16]. Enhanced blood flow associated with postural change from sitting to standing also increases delivery of oxygen, nutrients, and hormones, including insulin, to lower extremity tissues [17]. Furthermore, the regular activation of large muscles necessary to stand may improve functional status [18,19].

Metabolomic profiling enables observation of the physiological status of metabolic pathways through the quantification of low molecular weight metabolic intermediates and end products [20,21,22,23]. Mass spectrometry (MS)-based metabolomics has been largely used in many applications for disease diagnosis, drug development, and pathway analyses [24]. The plasma metabolome can reveal underlying pathology and molecular pathways involved in cardiometabolic disease risk [21,25,26,27]. Altered plasma concentrations of metabolic intermediaries of dysfunctional mitochondrial pathways have been shown to indicate insulin resistance and risk for T2D and CVD years before onset [28,29,30,31,32,33,34,35]. In addition, metabolomic analyses are able to reveal significant metabolic alterations associated with T2D, CVD, and cancer, as well as the complications of metabolic disease [36,37,38,39,40]. Currently, very few metabolomics studies have examined the metabolic signature of interrupting prolonged sitting [41,42].

Despite our knowledge of the health risks associated with prolonged sitting time, particularly in older adults, there are major mechanistic knowledge gaps in sitting intervention research as to why and what can be done to mitigate this risk. We aimed to use metabolomic profiling to elucidate the acute metabolic and physiological perturbations that occur during prolonged sitting with or without standing or walking breaks to reduce sitting bout length.

## 2. Methods

### 2.1. Study Overview

Archival plasma samples collected during a previously published pilot study were used for metabolomic profiling [17]. An abbreviated version of the protocol is provided here. The pilot study was a randomized, controlled, four-condition crossover trial approved by the University of California, San Diego (UCSD) Institutional Review Board (Approval Code: 150509, Approval Date: May 2016). Participants provided written informed consent, and this study was registered with Clinical Trials.gov (#NCT02743286). Participants completed each of the four conditions in randomized order.

### 2.2. Participants

Ten sedentary, overweight, or obese postmenopausal women aged fifty-five and older were randomized. Nine participants completed all four study visits. One participant was lost to follow up after completing three of four study visits. Exclusion criteria included poor glycemic control [(HbA1c) ≥ 53 mmol/mol (7%) in participants < 65 years of age or HbA1c ≥ 58 mmol/mol (7.5%) in participants ≤ 65 years of age], type 1 diabetes, anemia, insulin medication use, uncontrolled hypertension, personal or family history of venous thrombosis, tobacco or nicotine product use, five percent fluctuations in weight for the previous three months, donated blood less than 56 days prior to enrolling, weight influencing chronic illnesses, immunosuppressant or corticosteroid medication use, thrombosis risk (vasodilator medication use and/or a history of congestive heart failure, stroke, atrial fibrillation or 2+ hospitalizations within previous 6 months), currently enrolled in another study related to sedentary behavior or physical activity, the inability to walk for 5 min or stand in place for 10 min at a time, and/or the inability to rise independently from a seated position. Potential participants taking medications for type 2 diabetes and cardiovascular disease (with the exception of insulin) were permitted to enroll. Aggregated cardiometabolic medication use data are shown in Table 1.

### 2.3. Study Visits

Participants were consented to partake in all four conditions assigned in a computer-generated random order and to complete the conditions at separate clinic visits. Participants were blinded regarding visit conditions until their arrival for each visit. Between each condition, a seven-day wash-out period was required to diminish any carryover effects. Participants were asked to refrain from moderate to vigorous physical activity (MVPA) and alcohol use two days prior to the study visit. Participants were permitted to read (books, magazines, or newspapers), watch DVDs, perform light paperwork, and/or work on a laptop computer during study conditions. Participants were supervised by study staff during each visit to monitor and ensure condition protocol compliance. Clinical and circulating biomarker measurement methods have been described in detail previously [17].

**Figure 1:**
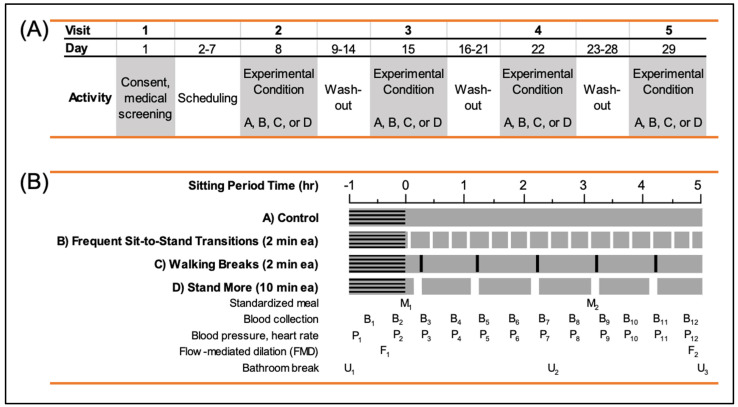
Schematic overview of clinic visits (**A**) and study protocol (**B**). Image adapted from figure shown in parent study paper [17].

Participants consumed a provided, standardized meal the night before each clinical visit (450–475 kcal, 23–25% fat, 52–56% carbohydrate, and 21–24% protein). Subsequently, participants were asked to fast for ten hours prior to their arrival by motor vehicle. Prior to the visit, required morning medications taken with water or medications taken with food during the clinic visit were permitted. Medication dosage was consistent for each subject during all visits. Participants were comfortably seated in a padded armchair. If the subject’s feet did not comfortably rest flat on the floor, footrests were provided. Blood sampling was taken via a catheter placed in the antecubital vein. An initial fasting blood draw (B1) was collected fifteen minutes following the catheter placement (condition period time −0.5 h) (Figure 1). Participants were asked to remain seated during a one-hour lead-in prior to the first meal. After the second fasting blood draw (B2) at the start of the condition period, participants were asked to consume the first standardized, liquid mixed meal (M1; condition period time 0 h). Blood samples were collected every 30 min thereafter. The liquid mixed meals (Ensure Plus^®^, 5 kcal/kg body weight) were fully consumed within 5 min at condition period times 0 h (M1) and 3 h (M2). The macronutrient composition of the Ensure Plus^®^ was 57% carbohydrate, 15% protein, and 28% fat to simulate a mixed meal, and water (8 oz) was provided after each meal intake. The mixed meals were administered with the goal of eliciting postprandial glucose and insulin responses across the experimental conditions that would be similar to those following real-world meals.

#### Condition Protocols

All four conditions began with a steady state period of one hour sitting, followed by a five-hour sitting condition, and a bathroom break at 2.5 h, which occurred in a private restroom fewer than twenty feet from the study room chair. The four study condition protocols were the following:

*Sit Control*: Participants were asked to sit quietly for five hours and to minimize excessive movement during the condition. This protocol was designed as the control condition for comparison in statistical analyses.

*Frequent Sit-to-Stand Transitions (STS)*: Participants were asked to stand two of every 20 min for the five-hour condition. Participants performed fifteen sit-to-stand transitions and thirty minutes of standing time across the condition. 

*Hourly Stands (Stand):* Participants were asked to stand for ten minutes once every hour. Participants stood on a padded floor mat. Small, brief stretching movements of the upper body were permitted without their lowering of the head below waist level. The protocol resulted in five sit-to-stand transitions and fifty minutes of standing time. 

*Hourly Walks (Walk)*: Participant completed a two-minute walk each hour throughout the condition. Participants were escorted by study staff in unobstructed hallways and encouraged to walk at a purposeful but comfortable pace. The protocol resulted in five sit-to-stand transitions and ten minutes of walking. 

### 2.4. Metabolomics

The primary metabolite assay was conducted at the University of California, Davis West Coast Metabolomics Center, to profile differences in primary metabolites comparing the baseline (B1) and final blood draw (B12) samples [43]. A targeted approach using LC-QTOF (HILIC) mass spectrometry was employed to focus on primary metabolism compounds, including a panel of 18 acylcarnitines and 296 metabolites.

#### 2.4.1. Sample Preparation

Samples were extracted using 1 mL of 3:3:2 ACN:IPA:H_2_O (*v*/*v*/*v*). Half of the collected sample was dried to completeness and then derivatized using 10 µL of 40 mg/mL of methoxyamine in pyridine. Samples were shaken for 1.5 h at 30 °C, then 91 µL of MSTFA + FAMEs were added to each sample and shook at 37 °C for 0.5 h to finish derivatization. Samples were then placed into a vial, capped, and injected onto the instrument.

#### 2.4.2. Gas Chromatography–Mass Spectrometry

A 7890A GC coupled with a LECO TOF platform was used to complete the analysis. 0.5 µL of derivatized sample was injected via a splitless method onto a Restek Corporation Rtx-5Sil MS (30 m length × 0.25 mm internal diameter with 0.25 µm film made of 95% dimethyl/5%diphenylpolysiloxane) column with an Intergra-Guard at 275 °C and a helium flow of 1 mL/min. The GC oven was set to hold at 50 °C for 1 min, then ramp to 20 °C/min to 330 °C, followed by holding for 5 min. The transfer line was set to 280 °C while the EI ion source was set to 250 °C. The mass spectrometer parameters collect data from 85 *m*/*z* to 500 *m*/*z* at an acquisition rate of 17 spectra/sec. Peaks from the GC-TOF MS platform were automatically detected and deconvoluted from coeluting peaks by the LECO ChromaTOF software (v3.0) [44].

#### 2.4.3. Data Processing

Apex masses were reported for use in the BinBase algorithm and implemented in the metabolomics BinBase database. The BinBase algorithm utilized the following settings: validity of chromatogram (<10 peaks with intensity > 10^7^ counts s^−1^), unbiased retention index marker detection (MS similarity > 800, validity of intensity range for high *m*/*z* marker ions), retention index calculation by 5th order polynomial regression. Spectra are cut to 5% base peak abundance and matched to database entries from most to least abundant spectra using the following matching filters: retention index window ±2000 units (equivalent to about ±2 s retention time), validation of unique ions and apex masses (unique ions must be included in apex masses and present at >3% of base peak abundance), mass spectrum similarity must fit criteria dependent on peak purity and signal/noise ratios, and a final isomer filter.

### 2.5. Statistical Analyses

Metabolite ratios were calculated as post-condition/baseline, and paired *t*-tests were used to examine between condition significance using Microsoft Excel (Redmond, WA, USA) and SPSS 29.0 (SPSS Inc., Chicago, IL, USA). Paired sample *t*-tests of the control condition and interventions were conducted for between-condition differences, and a Bonferroni correction (*p*-value/number of *t*-tests) was applied as a post-hoc procedure to reduce type 1 error. Orthogonal partial least squares-discriminant analysis (PLS-DA) and Variable Importance Projection (VIP) scores, pathway and enzyme enrichment analyses, and a heat map were log-transformed, auto-scaled, and analyzed between conditions using MetaboAnalyst software [45]. A threshold α-level of 0.05 was used to define statistical significance between groups, and a false discovery rate (q-value) was applied to the analyses.

## 3. Results

### 3.1. Participant Characteristics

Participant characteristics are shown for the 10 postmenopausal women randomized (Table 1). As described previously [17], the participants were primarily non-Hispanic White. On average, the participants had obesity and had central adiposity, as determined by waist circumference and waist/hip ratio. On average, they demonstrated impaired glucose regulation (determined by fasting plasma glucose ≥ 100 mg/dL and glycated hemoglobin (HbA1c) in the pre-diabetic range of ≥5.7%) and insulin resistance (determined by fasting plasma insulin > 7 μIU/mL and the Homeostatic Model Assessment for Insulin Resistance [HOMA-IR]). On average, the participants exhibited a normal heart rate and blood pressure.

### 3.2. Postprandial Metabolome Alterations during Experimental Sitting Conditions

Metabolome profiles from fasting (B1) and final post-condition (B12, 2-hour postprandial to Meal 2) plasma samples collected during the Control condition were compared. Paired *t*-tests of baseline and post-condition metabolite concentrations were conducted. The metabolome shift from a fasting to fed state was characterized by the significant lowering of fatty acids (Table 2).

Fold change ratios of post-condition abundance to fasting baseline abundance were used to evaluate within-condition effects on individual metabolites, with a value of 1.0 depicting no change. Metabolite within-condition changes significantly different between conditions are shown in Table 3. The frequent sit-to-stand (STS) condition significantly altered the postprandial levels of anserine, 5-hydroxynorvaline, γ-glutamylmethionine, glutamine, homoarginine, lysine, N-N-dimethylarginine, N-ε-Acetyllysine, cholesterol, and ε-Caprolactam compared to the Control condition. The STS effect was to primarily neutralize the elevated levels of these metabolites that were observed in the Control condition. The exceptions were 5-hydroxynorvaline and cholesterol, which were reduced in the Control condition but unchanged in the STS condition. The Walk condition significantly altered the postprandial levels of pipecolinic acid, palmitic acid, threitol, and azelaic acid compared to the Control condition. The Stand condition significantly altered the postprandial levels of aspartic acid, 5-hydroxynorvaline, and 2-deoxyguanosine monophosphate (2-dGMP) compared to the Control condition. The frequent sit-to-stand (STS) condition significantly altered the postprandial levels of several metabolites compared to the Walk and Stand conditions.

**Figure 2:**
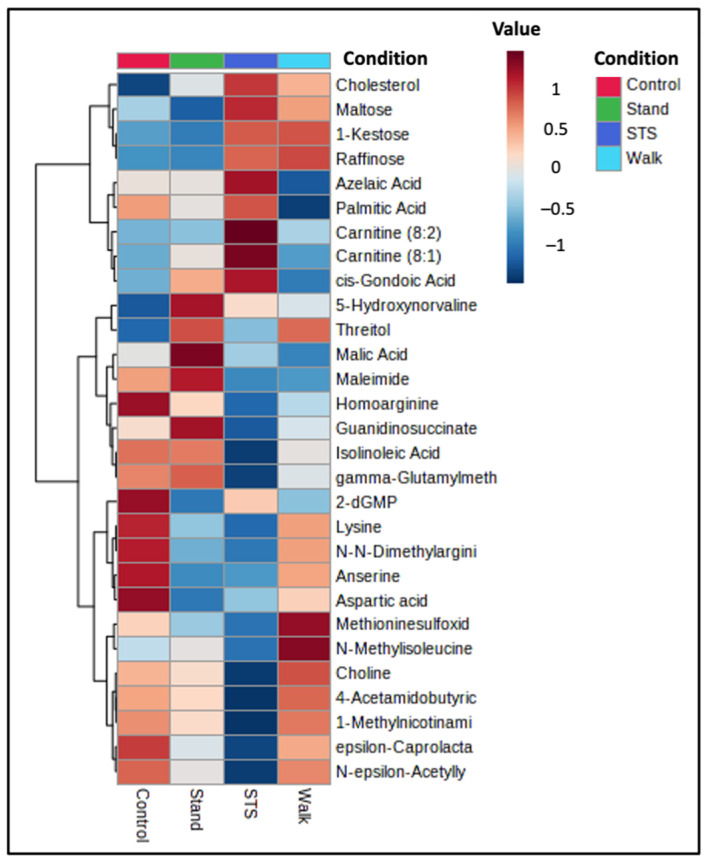
Heatmap of condition-averaged metabolites that were significantly different between postprandial conditions (*p* < 0.05). The display demonstrates normalized relative increases and decreases in abundance between and within conditions in postmenopausal women. N = 10 for the Control, Stand, and Walk conditions; N = 9 for the STS condition.

**Figure 3:**
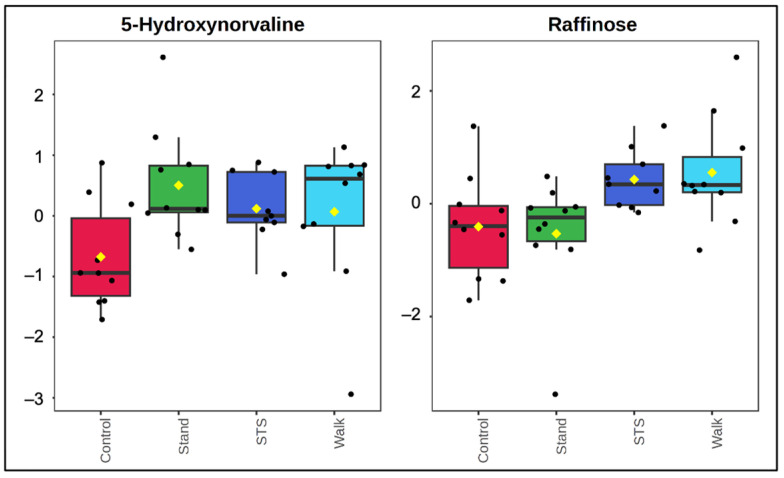
Mean average box plots of 5-hydroxynorvaline and raffinose across all conditions by post/pre-condition ratio of relative abundance. Black dots represent participant post/pre-condition ratio and yellow diamonds denote mean ratio relative abundance of each condition. N = 10 for the Control, Stand, and Walk conditions; N = 9 for the STS condition.

Post-hoc testing of between-intervention effects was performed and uncovered 19 significantly altered metabolites (Table S1). Volcano plots of each intervention and the control condition highlighting the significant changes between condition and fold change are provided in Figures S1–S3. Heatmaps showing condition-averaged and individual sample normalized mean relative change in abundance across the conditions were generated to illustrate the directionality of within- and between-condition change for each of the 29 significant metabolites (Figure 2 and Figure S4, respectively). Compared to the Control condition, all three sitting interruption modalities demonstrated unique and significant differences in plasma metabolite concentration patterns. Figure 3 shows boxplots for two of the metabolites listed in Figure 2. Boxplots for all metabolites listed in Figure 2 are shown in Figures S5 and S6.

**Figure 4:**
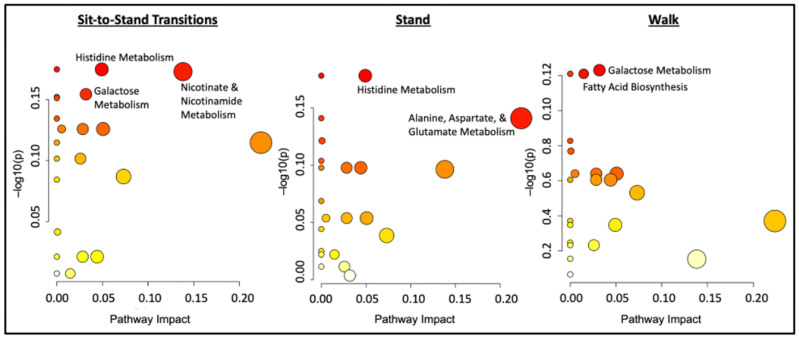
Pathway enrichment analysis of the three interrupted sitting conditions against the controlled sit. Data are plotted as −log_10_(*p*) versus pathway impact and darker color represent greater significant differences. N = 10 for the Control vs. Stand and Control vs. Walk conditions; N = 9 for the Control vs. STS condition.

**Figure 5:**
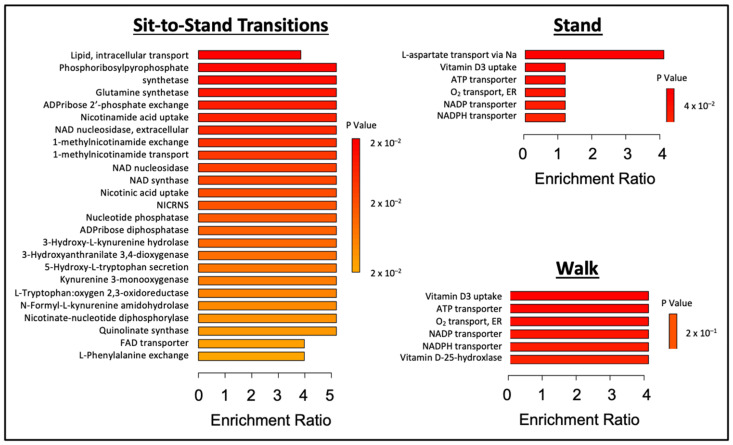
Enzyme enrichment analysis of the three sitting interruption conditions compared to the Control condition. N = 10 for the Control vs. Stand and Control vs. Walk conditions; N = 9 for the Control vs. STS condition.

Pathway and enzyme enrichment analyses are shown in Figure 4 and Figure 5, respectively. The STS and Control postprandial metabolomes exhibited significant differences in nicotinate and nicotinamide, histidine, and galactose metabolism. The Stand condition resulted in significantly different effects in histidine metabolism and alanine, aspartate, and glutamate metabolism compared to the Control condition. The Walk condition additionally resulted in significantly different effects in galactose metabolism and fatty acid biosynthesis compared to the Control condition.

**Figure 6:**
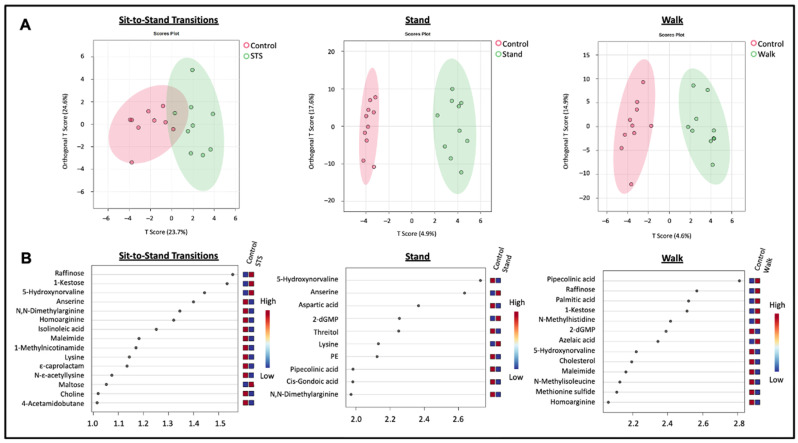
Orthogonal partial least squares-discriminant analysis (OPLS-DA) score plots (**A**) and variable importance projection (VIP) scores (**B**). A. OPLS-DA plots indicate discrete condition-specific postprandial metabolomes. B. VIP score-ranked metabolites indicate the most influential metabolites distinguishing the interrupted sitting modality from the Control in the OPLS-DA models. N = 10 for the Control vs. Stand and Control vs. Walk conditions; N = 9 for the Control vs. STS condition.

In Figure 6, orthogonal partial least squares-discriminant analyses (OPLS-DA) were conducted between each sitting interruption condition and the Control condition with variable importance projection (VIP) scores denoting significant drivers of the models (VIP > 1.0). The PLS-DA models highlight the within-condition similarities and the significant differences between the sitting interruption conditions and the Control condition.

## 4. Discussion

We employed a targeted metabolomics approach to investigate the postprandial metabolome and better understand the physiological benefits of varied modalities of interrupting prolonged sitting in postmenopausal women with cardiometabolic risk. The hormonal shifts of menopause place women at an increased risk for several cardiometabolic conditions [9]. Moreover, sitting for long uninterrupted bouts compounds the susceptibility of developing obesity and the same conditions due to reduced hemodynamics and endothelial dysfunction [3,10,12]. Practical methods that reduce extended sitting bouts and are also effective in combating the risk of developing chronic conditions are needed for improved postmenopausal health.

To our knowledge, this is the first postprandial metabolomic analysis of a meal tolerance test during a controlled five-hour seated condition in postmenopausal women. We aimed to explore how interrupting prolonged sitting with three different postural change modalities alters that postprandial metabolome using paired plasma samples from a pilot randomized controlled crossover trial. Our findings in the Control condition yielded expected fasting-to-fed metabolite changes. Concentrations of fatty acids and glycerol observed at baseline as a result of fasting-associated adipocyte lipolysis were later suppressed at the postprandial time point, as postprandial insulin shuts down lipolysis. The consumption of the mixed meals also yielded predictable postprandial increases in amino acid concentrations.

The STS condition was distinctive in the number of significant differences in postprandial metabolites compared to the Control condition vs. the other interruption modality conditions. The differences likely reflect the frequency of postural changes and energy expenditure required. The 2-hour postprandial levels of amino acids and their derivatives were unchanged from fasting in the STS condition, which was significantly different from the large increases in these compounds during the Control condition. This postprandial metabolome difference may indicate the ability of frequent postural changes to enhance blood flow throughout the condition, which will enhance metabolism, delivery of amino acids to tissues, and/or absorption of mixed meal nutrients. The observed increased clearance of plasma amino acids during the STS condition is similar to the reported metabolome changes in amino acids observed in postmenopausal women after moderate-to-vigorous physical activity (MVPA) [41] and cross-sectionally associated with MVPA in a population of older adults [46]. The similarity of our STS postprandial metabolome with these MVPA results may potentially be due to the relative energy expenditure increase for our overweight/obese postmenopausal sample. The cardiometabolic benefits of merely rising to stand improve blood flow and oxygen and nutrient delivery to the digestive tract, peripheral tissues, and skeletal muscle [15,16,17]. Regular postural changes also require large muscle contractions and increase energy expenditure to rise and stay in a standing position [18,19]. Interestingly, circulating cholesterol was slightly reduced after the controlled sit but nearly unchanged after the STS visit, a difference between conditions that was statistically significant.

The STS condition postprandial metabolome was also remarkable for pathways related to endothelial and mitochondrial functioning as well as gut permeability. Previous studies have shown the impact of histidine derivatives, such as beta-alanine-containing compounds carnosine and anserine, on increasing nitric oxide production in endothelial cells and improving blood flow [47]. Their supplementation increases blood flow via endothelial relaxation and increases insulin-stimulated glucose uptake in skeletal muscle [48,49,50,51]. Pathway analysis of the STS plasma metabolome revealed altered histidine metabolism driven by lower postprandial levels of anserine than the Control potentially due to depletion from increased nitric oxide activity. The maintenance of nicotinamide adenine dinucleotide plus hydrogen (NADH) delivery to the mitochondria in the skeletal muscle for adenosine triphosphate (ATP) production is primarily through the malate-aspartate shuttle, which is necessary during increased physical activity [52]. The analysis also uncovered modified nicotinate and nicotinamide metabolism as a result of decreases in 1-methylnicotinamide and aspartate after the STS condition. Enzyme enrichment analysis also highlighted an increased metabolic demand through elevated lipid intracellular transport and NAD uptake, exchange, and transport. The STS condition had significantly different effects on galactose metabolism due to postprandial increases in plasma raffinose, a trisaccharide composed of glucose-bridging galactose and fructose digestible only by gut microbes. Previous studies report a rise in circulating raffinose after ingestion during physical activity due to a proposed induced state of gut permeability [53,54].

Both the STS and hourly stand (Stand) conditions had significantly different postprandial changes in the compound 5-hydroxynorvaline compared to the Control condition. The hourly stand condition had a nearly two-fold postprandial concentration change in 5-hydroxynorvaline (1.72, *p* = 0.011 vs. Control); the postprandial concentration was practically unchanged during the STS condition (0.94, *p* = 0.048 vs. Control) in compared to that of the Control (0.71). An isomer of the branched chain amino acid valine and derivative of norvaline, 5-hydroxynorvaline is a powerful inducer of nitric oxide that has been shown to have blood pressure and blood sugar-lowering effects [55,56]. The relative increase in the postprandial levels of 5-hydroxynorvaine during the Stand condition compared to Control closely aligns with the significant physiological improvement in flow-mediated dilation (FMD) observed after the Stand condition in the parent study (*p* = 0.033 vs. Control). The Stand condition ten-minute hourly stands also increased aspartic acid and decreased 2-deoxyguanosine monophosphate (2-DGMP). Aspartic acid is also one of the building blocks of proteins, and its presence in the plasma is typically a result of protein catabolism, while 2-DGMP is a DNA monomer nucleotide [52,57]. The pathway and enzyme enrichment analyses similarly found altered histidine metabolism and alanine, aspartate, and glutamate metabolism due to lower concentrations of anserine and aspartate as well as the enzyme responsible for aspartate transport via sodium compared to the Control condition.

The Walk two-minute hourly walking condition found higher postprandial elevations of pipecolinic acid, the major intermediate in lysine degradation in the mitochondria, and the sugar substitute threitol [58], compared to the Control condition. The Walk condition also resulted in significantly lower postprandial plasma concentrations of azelaic acid, potentially due to its ability to induce mitochondrial biogenesis in the skeletal muscle [59], and palmitic acid compared to the control. Pathway analysis also revealed an effect on fatty acid biosynthesis, which was driven by a greater postprandial concentration of palmitic acid in the controlled sit. Both conditions reported reduced levels post condition, most likely as lipolysis declined, but the hourly walks proved to have a greater lowering effect. Palmitic acid is the most common saturated fatty acid and is associated with several chronic conditions like atherosclerosis, coronary artery disease, and T2D [60]. The hourly walking breaks appear to have helped combat this negative effect of prolonged sitting. The Walk condition also resulted in elevated circulating raffinose levels compared to the Control condition, potentially due to activity-enhanced gut permeability as observed with the STS intervention and prior research in MVPA [53,54]. The enzyme enrichment analysis did not detect any significant differences between conditions. This commonality in the STS and Walk intervention postprandial metabolomes may be attributed to the relatively greater energetic demand of the two sitting interruption modality conditions in contrast with the hourly Stand intervention and Control condition.

Orthogonal PLS-DA modeling of the sitting interruption modality and Control conditions confirms between-condition differences observed with other comparison analyses. Appearing with VIP scores greater than 2.2 for all interventions, 5-hydroxynorvaline was the most significant driver for the Stand condition postprandial metabolome (VIP = 2.7); its vasoactive function was described above. The STS and Walk OPLS-DA models were largely driven by trisaccharides, raffinose and 1-kestose, a fructooligosaccharide, which are undigestible by human enzymes but which can be fermented by gut microbes [61,62]. The models demonstrate comparable separation of the interventions and control, yet still distinctly illustrate how each sitting interruption modality may potentially provide unique, acute postprandial metabolic alterations.

This study was a secondary analysis of plasmas collected during a previously completed trial and has notable limitations. It was not powered to identify significant alterations in postprandial metabolite concentrations. Despite the crossover design, a relatively small sample size of predominantly White, non-Hispanic postmenopausal women was used; thus, results may not be generalizable to other populations. Our prior work suggests that Hispanic postmenopausal women have an increased risk of poor glycemic control associated with prolonged sitting time [63] and similarly may have ethnically distinct metabolomic profiles associated with postprandial sitting time. This analysis was also an opportunistic sampling of the 0-hour and 5-hour timepoints. Ideally, all timepoints across the 5-hour conditions would be assessed for a more comprehensive depiction of postprandial flux in the metabolome. We did not collect time since menopausal transition, which may influence outcomes. We estimate the mean time since menopausal transition to be eleven years given our inclusion criteria. Very low estrogen status associated with menopause is associated with a risk of systemic inflammation and may be an effect modifier of our outcomes. Given the small size of this pilot exploratory secondary metabolomics study, we do not have statistical power to explore moderation effects of medication on postprandial metabolome. Future work may consider participant inflammatory status and medication use as potential effect moderators. Additional research testing biologically impactful modalities for interrupting prolonged sitting is needed. Follow-up sample analyses of a recently completed, large-scale trial (N = 79 enrolled) will be integral in validating these results (NCT03511352).

## 5. Conclusions

In this pilot crossover design study, we employed a targeted metabolomics approach to investigate the postprandial metabolome in postmenopausal women with overweight or obesity during a prolonged sitting condition. We further evaluated the impact of three different sitting interruption modalities on the postprandial metabolome. Compared to the Control condition, we observed significant improvements in amino acid metabolism (absorption, clearance, and/or catabolism) after the frequent sit-to-stand condition (STS) and metabolite patterns indicative of improved endothelial health during the hourly standing condition (Stand). A significant reduction in free fatty acids was also noted with the two-minute hourly walks (Walk) condition in comparison with the Control condition. Our findings contribute to the growing literature demonstrating the beneficial effects of breaking up prolonged sitting and the unique health impacts of different interruption modalities.

## Figures and Tables

**Table 1:** Participant demographics and clinical characteristics.

Measurement	
*N*	10
Age (y)	66 ± 9.0
BMI (kg/m^2^)	30.6 ± 4.2
Body mass (kg)	79.4 ± 12.3
Height (cm)	161 ± 5.9
Waist circumference (cm)	95.9 ± 11.8
Hip circumference (cm)	104.5 ± 15.2
Waist/hip ratio	0.92 ± 0.07
HbA1c (%)	5.7 ± 0.5
HbA1c (mmol/mol)	39 ± 6.0
Fasting plasma glucose (mg/dL)	107.2 ± 17.4
Fasting plasma insulin (uIU/mL)	9.3 ± 4.8
HOMA-IR	2.5 ± 1.5
Systolic BP (mmHg)	123 ± 8.0
Diastolic BP (mmHg)	66 ± 7.0
Heart rate (bpm)	65 ± 8.0
**Race**	**Count**
White	9
Asian	1
**Ethnicity**	
Hispanic	2
Non-Hispanic	8
**Medications**	
β-blockers	1
β-3 adrenergic agonists	1
Calcium channel blockers	1
Loop diuretics	1
Metformin	2
Statins	2

Note: All non-count data are represented as mean ± standard deviation. BMI—body mass index; HbA1c—glycated hemoglobin; HOMA-IR—Homeostatic Model Assessment of Insulin Resistance; and BP—blood pressure.

**Table 2:** Fold change of prolonged sitting metabolites.

Metabolite	Fold Change	*p*-Value
Palmitoleic acid	0.14	<0.001
Palmitic acid	0.64	<0.001
Oleic acid	0.17	<0.001
Myristic acid	0.35	<0.001
Linolenic acid	0.41	<0.001
Linoleic acid	0.34	<0.001
Heptadecanoic acid	0.55	<0.001
Arachidonic acid	0.63	<0.001
Glycerol	0.67	<0.001

Note: Fold change was calculated as postprandial/fasting concentration. Paired *t*-tests of baseline and post-Control condition metabolite concentrations were conducted. The Bonferroni-corrected cut-off for significance was *p* < 0.002. N = 10.

**Table 3:** Fold change and paired samples *t*-tests comparing interventions with control.

Metabolites	Control v.		
	Sit-to-Stand	Stand	Walk
*Amino Acid Metabolism*			
Anserine	0.048 (1.27, 0.72)		
Aspartic acid		0.047 (1.72, 1.87)	
5-Hydroxynorvaline	0.048 (0.71, 0.94)	0.011 (0.71, 1.72)	
γ-Glutamylmethionine	0.020 (1.43, 0.97)		
Glutamine	0.042 (1.13, 0.97)		
Homoarginine	0.030 (1.57, 0.95)		
Lysine	0.033 (1.62, 1.10)		
N-N-Dimethylarginine	0.044 (1.30, 0.88)		
N-ε-Acetyllysine	0.026 (1.65, 1.14)		
Pipecolinic Acid			0.016 (0.69, 1.09)
*Fat Metabolism*			
Cholesterol	0.047 (0.89, 0.98)		
Palmitic Acid			0.028 (0.64, 0.50)
*Sugar Metabolism*			
Threitol			0.020 (0.99, 1.19)
*Other Organic Compounds*			
Azelaic Acid			0.043 (0.72, 0.58)
ε-Caprolactam	0.018 (1.60, 0.83)		
2-dGMP		0.028 (1.49, 0.72)	

Notes: *p*-Values were determined using paired sample *t*-tests. Ratios in parentheses were calculated as post/pre-concentration. Values > 1.0 were increased at end of condition. Ratios are listed in their respective order (control, intervention), and FDR q-values were considered significant at <0.05. N = 10 for Stand and Walk comparisons with Control; N = 9 for the STS comparison with Control.

## Data Availability

The original contributions presented in this study are included in this article; further inquiries can be directed to the corresponding author.

## Supplementary Materials

The following supporting information can be downloaded at: https://www.mdpi.com/article/10.3390/metabo14090478/s1, Figure S1: Volcano plot of STS and Control Conditions; Figure S2: Volcano plot of Stand and Control Conditions; Figure S3: Volcano plot of Walk and Control Conditions; Figure S4: Heat Map of All Conditions and Samples; Figure S5: Box Plots of All Significant Metabolites between interrupting sitting modalities and the Control condition; Figure S6: Box Plots of All Significant Metabolites between interrupting sitting modalities; Table S1: Significant Metabolites Resulting from Between-Condition Comparisons of Sitting Interruption Modalities.
