# Supplementary material for: Sitting Interruption Modalities during Prolonged Sitting Acutely Improve Postprandial Metabolome in a Crossover Pilot Trial among Postmenopausal Women
Source: Metabolites. 2024 Aug 30;14(9):478. doi: 10.3390/metabo14090478 (PMC11433994; doi:10.3390/metabo14090478)
Supplement: Supplementary file 1 [file metabolites-14-00478-s001.zip › metabolites-3152713-supplementary.pdf]

Supporting Information

Sitting Interruption Modalities During Prolonged Sitting Acutely Improve Postprandial Metabolome in Crossover Pilot Trial Among Postmenopausal Women

Jeffrey S. Patterson[1], Brinda K. Rana[2], Haiwei Gu[1], Dorothy D. Sears*[1,3,4,5]

Affiliations:
[1]College of Health Solutions, Arizona State University, Phoenix, AZ 85004, USA
[2]Department of Psychiatry, UC San Diego, La Jolla, CA, 92093
[3]Department of Family Medicine and Public Health, UC San Diego, La Jolla, CA 92093, USA
[4]Department of Medicine, UC San Diego, La Jolla, CA 92093, USA
[5]UCSD Moores Cancer Center, 3855 Health Sciences Dr, La Jolla, CA 92093 USA

Contents:

1. Supplementary Table S1: Significant Metabolites Resulting from Between-Condition Comparisons of Sitting Interruption Modalities

2. Supplementary Figure S1: Volcano plot of STS and Control Conditions
3. Supplementary Figure S2: Volcano plot of Stand and Control Conditions
4. Supplementary Figure S3: Volcano plot of Walk and Control Conditions
5. Supplementary Figure S4: Heat Map of All Conditions and Samples
6. Supplementary Figure S5: Box Plots of All Significant Metabolites between interrupting sitting modalities and the Control condition
7. Supplementary Figure S6: Box Plots of All Significant Metabolites between interrupting sitting modalities

Table S1. Significant Metabolites Resulting from Between-Condition Comparisons of Sitting Interruption Modalities

Table S1. Fold Change and Paired Samples t-tests of Sitting Interruption Modalities

**Metabolites**	**Sit-to-Stand v. Walk**	**Sit-to-Stand v. Stand**	**Walk v. Stand**
Amino Acid Metabolism			
**4-Acetamidobutyric acid**		0.046 (0.00, 0.03)	
**γ-Glutamylmethionine**	0.037 (0.97, 1.65)	0.033 (0.97, 1.52)	
**Maleimide**		0.034 (0.80, 1.30)	0.020 (0.77, 1.30)
**Methionine sulfoxide**			0.028 (1.64, 1.51)
**N-N-Dimethylarginine**			
**N-ε-Acetyllysine**	0.040 (1.14, 1.48)		
**N-Methylisoleucine**	0.019 (0.92, 1.28)		
Fat Metabolism			
**Carnitine (8:1)**	0.035 (1.73, 0.93)		
**Carnitine (8:2)**	0.035 (1.46, 1.07)		
**cis-Gondoic Acid**			0.049 (0.34, 0.44)
**Isolinoleic Acid**		0.048 (0.90, 1.39)	
Sugar Metabolism			
**Guanidinosuccinate**		0.028 (0.89, 1.21)	
**1-Kestose**		0.006 (1.25, 0.52)	
**Malic Acid**			0.032 (0.90, 0.94)
**Maltose**		0.044 (1.39, 0.56)	
**1-Methylnicotinamide**	0.007 (0.87, 1.43)	0.021 (0.87, 1.29)	
**Raffinose**		0.016 (1.28, 0.53)	
Other Organic Compounds			
**ε-Caprolactam**	0.048 (0.83, 1.33)		
**Choline**	0.039 (0.87, 2.43)		

Note. *P*-Values were determined by paired sample t-tests and ratios in parentheses were calculated as Post/Pre condition. Values >1.0 were increased at end of condition. Ratios are listed in their respective order and *P*-Values were considered significant at <0.05. N = 10 for the Stand and Walk conditions; n = 9 for the STS condition.

Supplementary Figure S1:

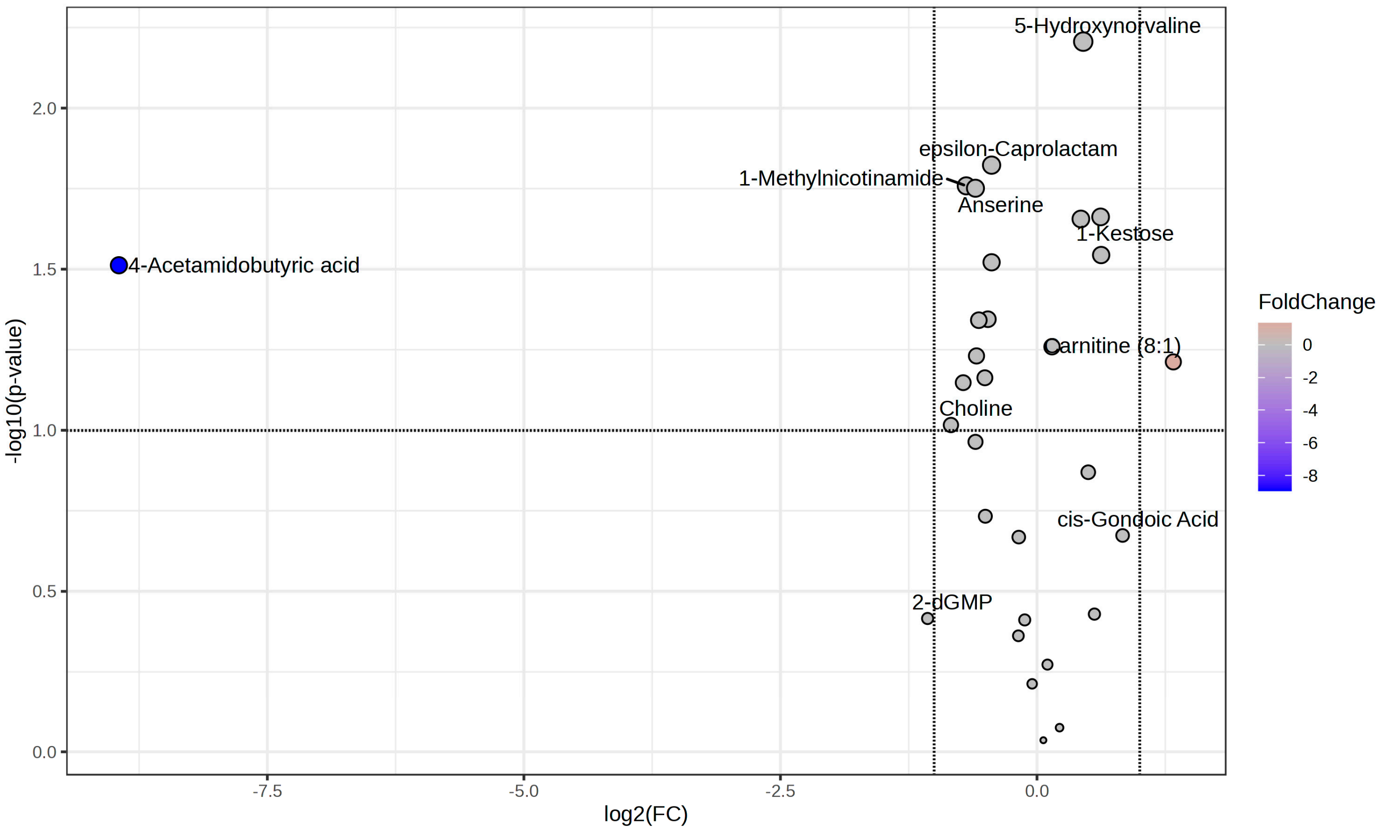

Figure S1. Volcano plot of STS and Control Conditions. Fold change calculated as STS / Control. N = 9.

Supplementary Figure S2:

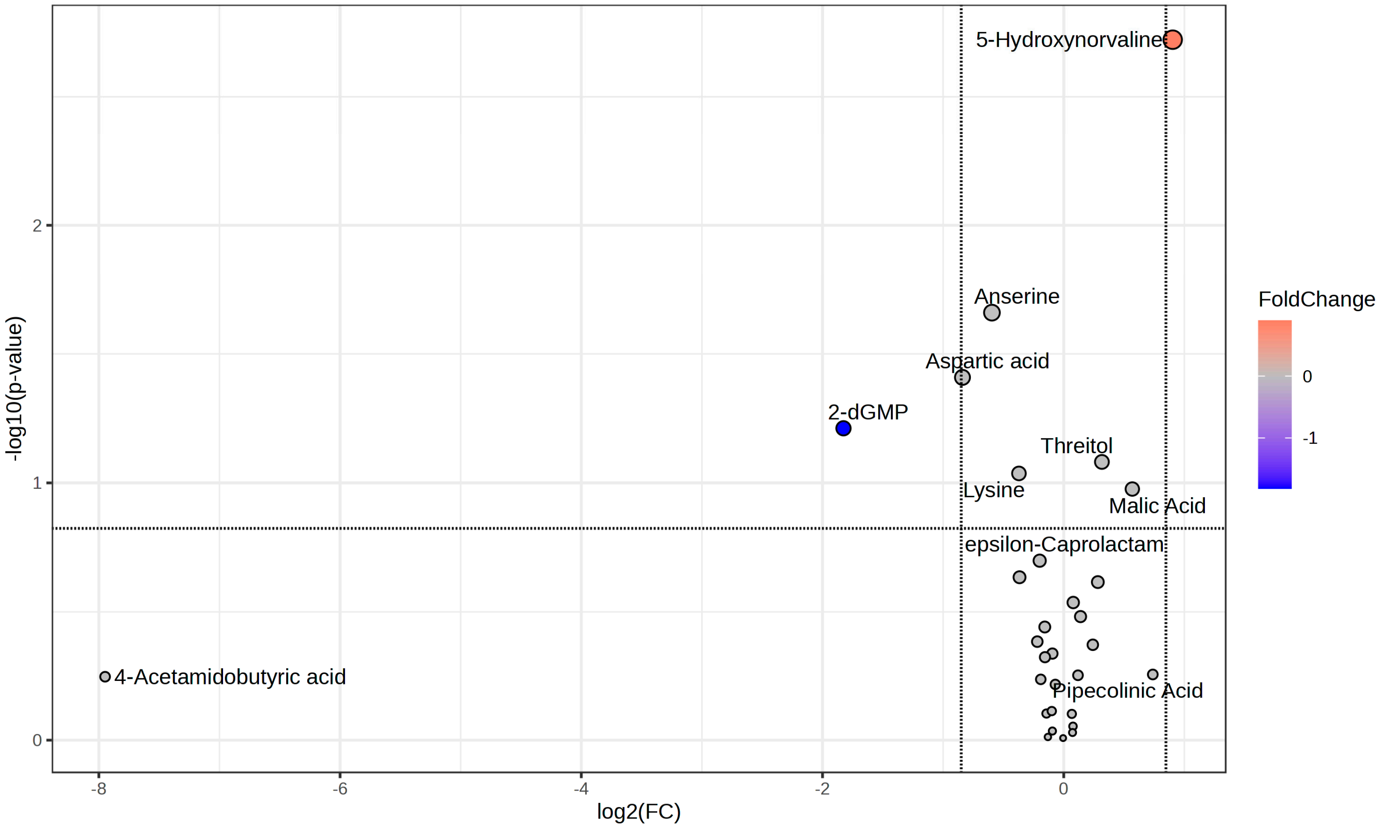

Figure S2. Volcano plot of Stand and Control Conditions. Fold change calculated as Stand / Control. N = 10.

Supplementary Figure S3:

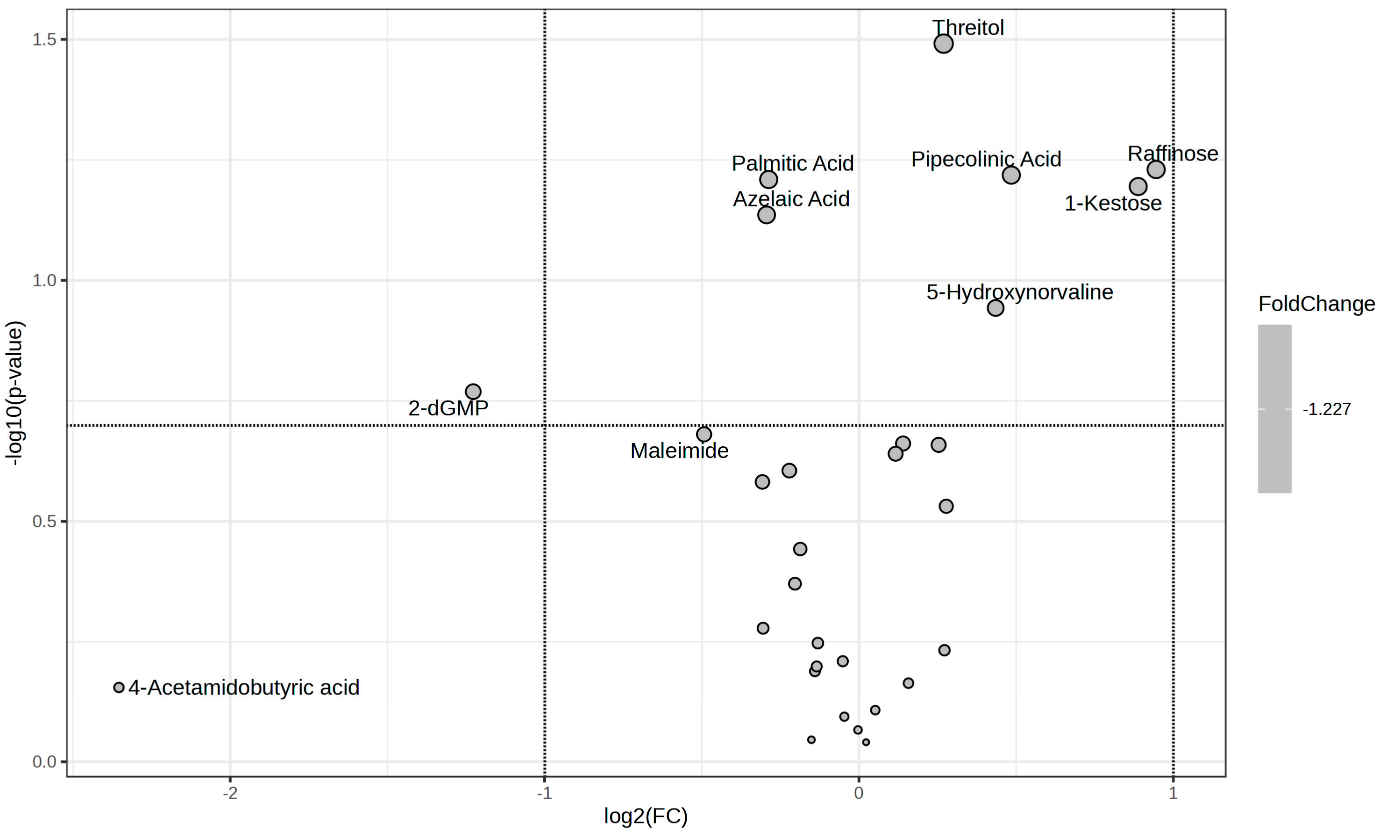

Figure S3. Volcano plot of Walk and Control Conditions. Fold change calculated as Walk / Control. N = 10.

Supplementary Figure S4

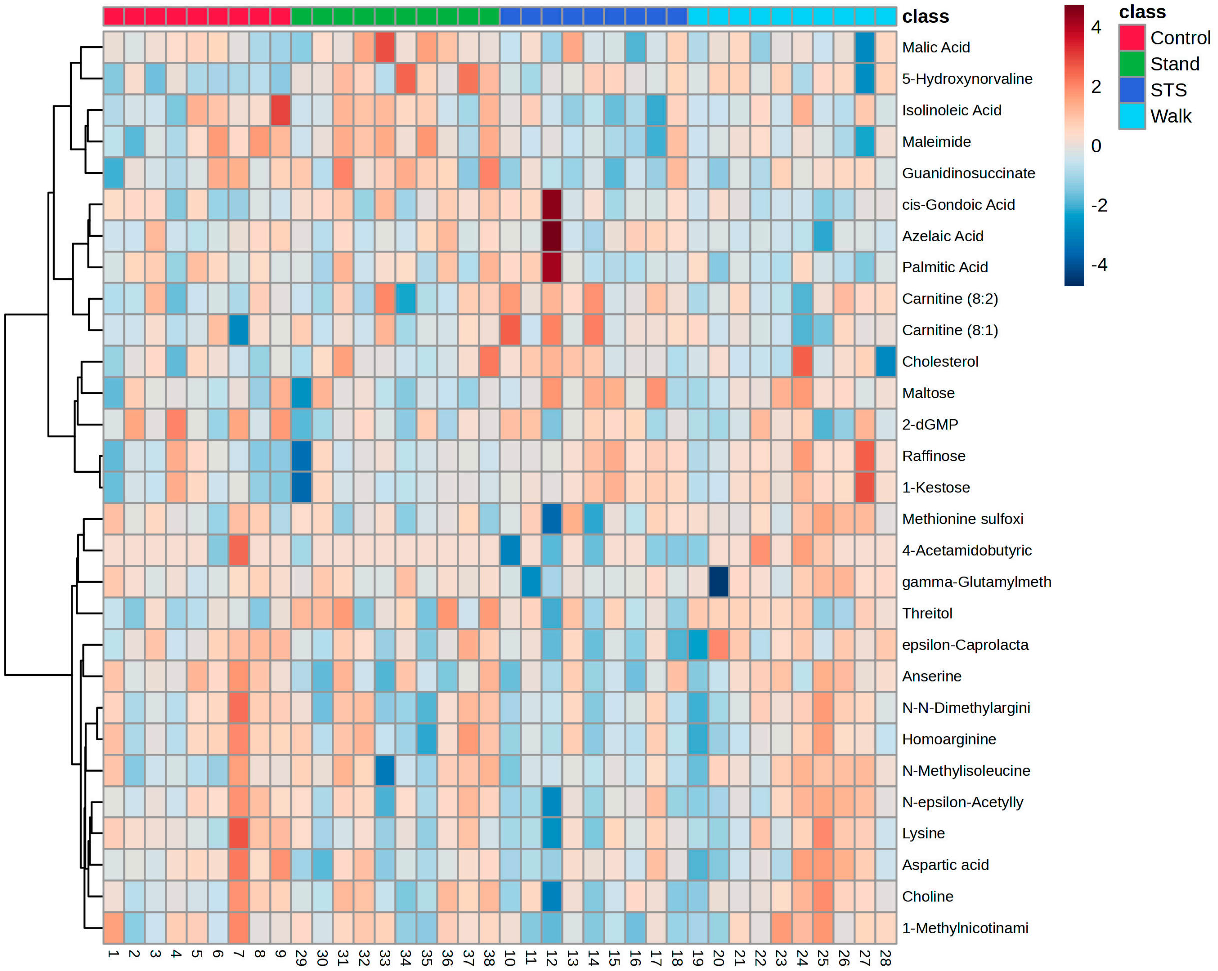

Figure S4. Heat Map of All Conditions and Samples. N = 10 for the Control, Stand, and Walk conditions; n = 9 for the STS condition.

Supplementary Figure S5:

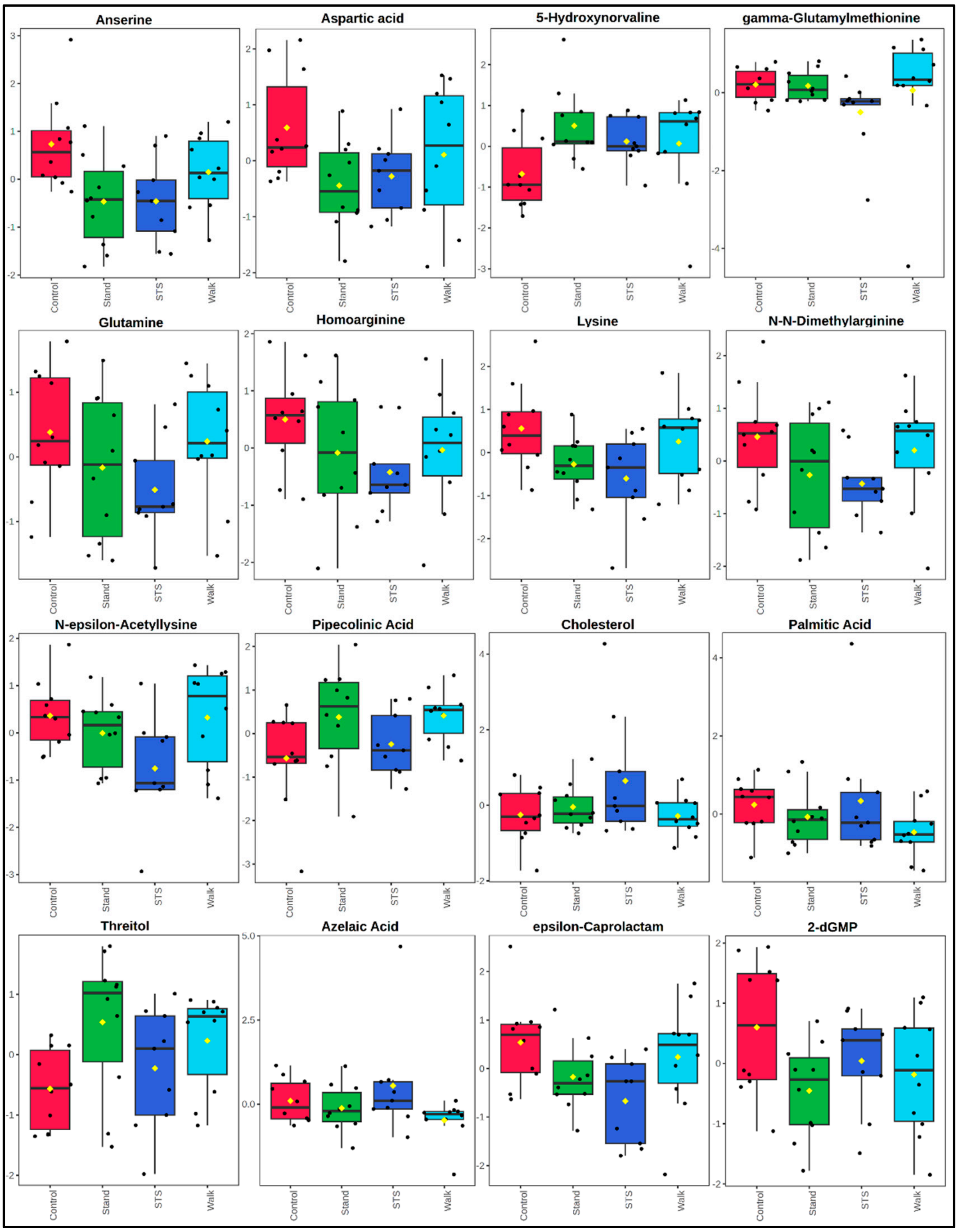

Figure S5. Box plots of all metabolites significantly different between sitting interruption modalities and the Control condition by Post/Pre condition ratio of relative abundance. N = 10 for the Control, Stand, and Walk conditions; n = 9 for the STS condition.

Supplementary Figure S6:

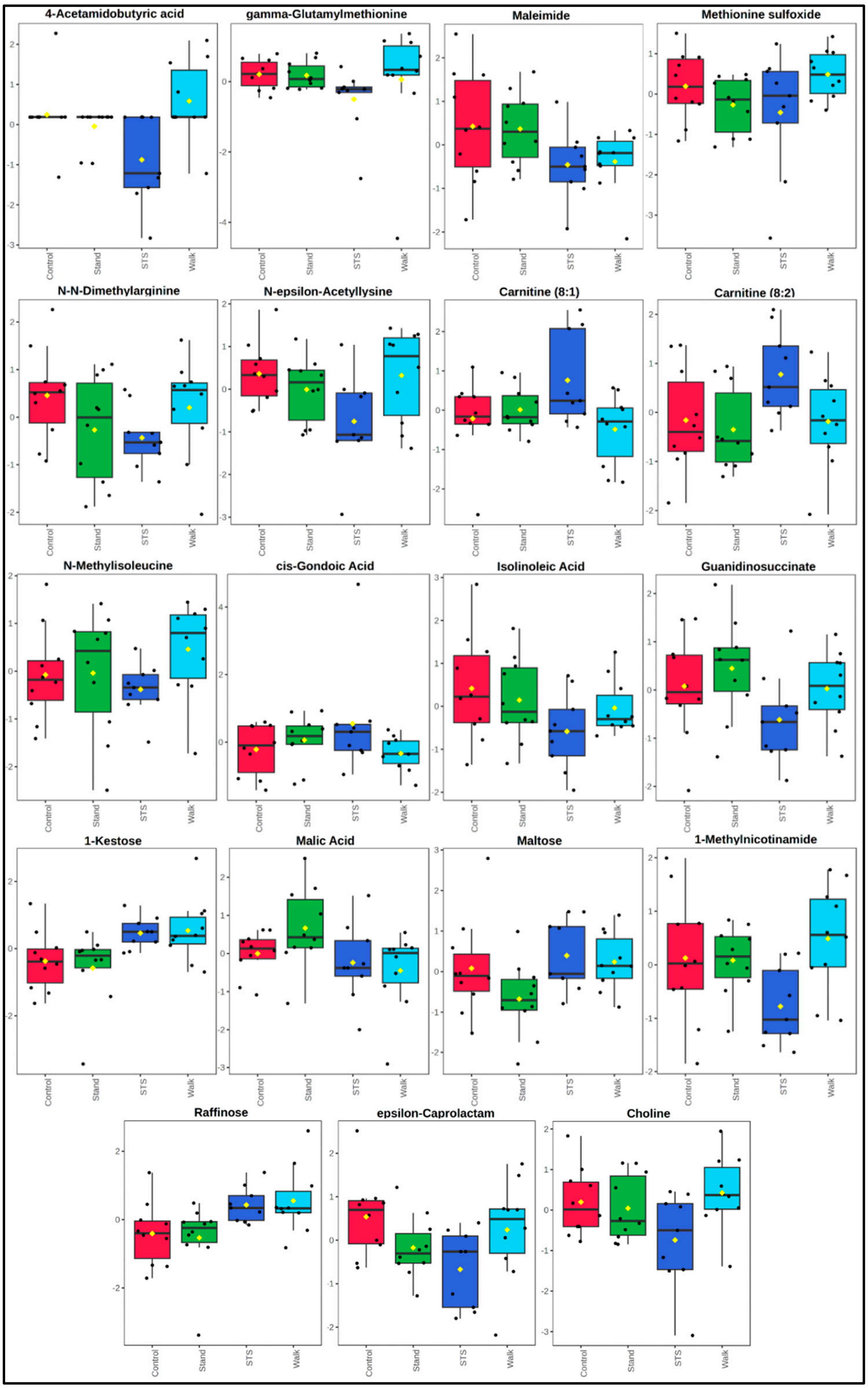

Figure S6: Box plots of all metabolites significantly different between sitting interruption modalities by Post/Pre condition ratio of relative abundance. N = 10 for the Control, Stand, and Walk conditions; n = 9 for the STS condition
